# Individualization of Duration of Dual Antiplatelet Therapy after Coronary Stenting: A Comprehensive, Evidence-Based Review

**DOI:** 10.3390/jcm12227144

**Published:** 2023-11-17

**Authors:** Gabriele Carciotto, Francesco Costa, Victoria Garcia-Ruiz, Mattia Galli, Emmanuele Soraci, Alberto Magliarditi, Lucio Teresi, Enrica Nasso, Scipione Carerj, Gianluca Di Bella, Antonio Micari, Giuseppe De Luca

**Affiliations:** 1 Division of Cardiology, Policlinico G Martino, 98125 Messina, Italy; gcarciotto97@gmail.com (G.C.); lucioteresi@gmail.com (L.T.); 2 BIOMORF Department, University of Messina, 98122 Messina, Italy; dottfrancescocosta@gmail.com (F.C.); antonio.micari@unime.it (A.M.); 3 Division of Cardiology, GIOMI Hospital, 98165 Messina, Italy; mavigaru@gmail.com; 4 Maria Cecilia Hospital, GVM Care & Research, 48033 Cotignola, Italy; dottormattiagalli@gmail.com; 5 U.O.S. Emodinamica, Department of Medicine, Ospedale Barone Romeo di Patti, 98066 Messina, Italy; manu.soraci@gmail.com (E.S.); albertomagliarditi@gmail.com (A.M.); 6 Department of Clinical and Experimental Medicine, University of Messina, 98122 Messina, Italy; enrica@nasso.it (E.N.); scarerj@unime.it (S.C.); gianluca.dibella@unime.it (G.D.B.); 7 Division of Cardiology, IRCCS Hospital Galeazzi-Sant’Ambrogio, 20157 Milan, Italy

**Keywords:** dual antiplatelet therapy, percutaneous coronary intervention, acute coronary syndrome, bleeding risk, tailored therapy

## Abstract

Dual antiplatelet therapy (DAPT), comprising aspirin and a P2Y12 receptor inhibitor, is the cornerstone of post-percutaneous coronary intervention treatment to prevent stent thrombosis and reduce the risk of adverse cardiovascular events. The selection of an optimal DAPT regimen, considering the interplay of various antiplatelet agents, patient profiles, and procedural characteristics, remains an evolving challenge. Traditionally, a standard duration of 12 months has been recommended for DAPT in most patients. While contemporary guidelines provide general frameworks, DAPT modulation with longer or shorter treatment courses followed by aspirin or P2Y12 inhibitor monotherapy are evolving towards an individualized strategy to optimize the balance between efficacy and safety. This review comprehensively examines the current landscape of DAPT strategies after coronary stenting, with a focus on emerging evidence for treatment individualization.

## 1. Introduction to the Rationale of Antiplatelet Therapy after Coronary Stenting

Percutaneous coronary intervention (PCI), especially with the availability of new drug-eluting stent (DES) technologies and new devices and drugs, has revolutionized the management of coronary artery disease (CAD) by providing effective revascularization and improving clinical outcomes [1,2,3,4,5,6]. Despite these achievements, CAD still represents the leading cause of mortality in developed countries [7,8] and the outcome is still unsatisfactory in high-risk patients [9,10,11]. Therefore, great attention has been paid so far to the identification of new risk factors [12,13,14,15] and implementation of primary and secondary prevention [16,17,18,19]. Antiplatelet therapies represent a keystone in secondary cardiovascular prevention. In fact, dual antiplatelet therapy (DAPT), comprising aspirin and a P2Y12 receptor inhibitor, is a cornerstone therapy in both elective and Acute Coronary Syndrome (ACS) patients treated by PCI and stenting, to prevent both stent thrombosis (ST) and ischemic events of other vascular segments, as it is well known that platelet adhesion, activation, and aggregation play a crucial role in the pathogenesis of vascular thrombosis [16].

**Figure 1:**
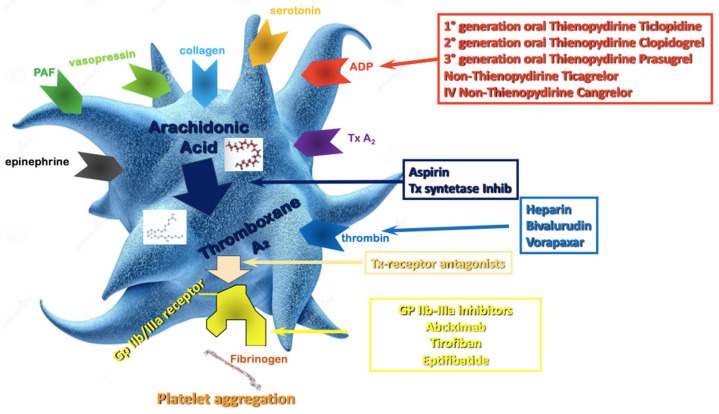
Antiplatelet therapy treatment pathways. ADP—Adenosine Diphosphate; Tx—Thromboxane; PAF—Platelet Activating Factor; Gp—Glycoprotein.

Aspirin is still widely considered the “primary agent” for the treatment of acute or chronic coronary syndrome to which additional antiplatelet drugs are added when a more intense antithrombotic effect is needed (Figure 1). Its antithrombotic action is based on the acetylation of the platelet’s cyclooxygenase (COX) [20], inhibiting the thromboxane A2 pathway. The interaction between ADP and the platelet P2Y12 receptor is another essential part of the platelet’s activation process, causing enhanced platelet degranulation and thromboxane production, and prolonged platelet aggregation [21]. The inhibition of the P2Y12 pathway prevents the binding of ADP to the receptor, attenuating platelet aggregation. In patients with acute coronary syndrome (ACS) or undergoing PCI for chronic coronary syndrome (CCS), DAPT has been shown to reduce recurrent major adverse cardiovascular events (MACE) compared to aspirin alone [22,23]. 

However, the anti-thrombotic benefits of DAPT are counterbalanced by an increase in bleeding, which is directly related to the duration and intensity of the antiplatelet regimen, and significantly impact mortality with similar time-dependency of myocardial infarction [24]. Hence, determining the optimal duration and selection of antiplatelet agents for DAPT is of the utmost importance to optimize clinical outcomes and remains a subject of ongoing investigation and clinical debate [25,26]. Recent studies have challenged the traditional notion of a one-size-fits-all approach, suggesting that individualized treatment strategies based on patient-specific factors and procedural characteristics may be necessary to select treatment [27]. Nowadays, the number of options to personalize DAPT treatment is vast, and those options are increasingly endorsed by international guidelines, leveraging on the modulation of DAPT duration (i.e., short vs. long treatment courses) [28], DAPT components in term of P2Y12 inhibitor type (i.e., potent vs. non-potent P2Y12 inhibitor), and dosage [29]. The aim of this review is to summarize the evidence that, over the years, has established the current options for DAPT modulation after PCI. 

## 2. Dual Antiplatelet Therapy and Choice of the P2Y12 Inhibitor

The CURE trial [16] established clopidogrel as the standard of care for DAPT after ACS or PCI. This was a randomized, double-blind, placebo-controlled trial with the aim to test the safety and efficacy of clopidogrel (loading dose of 300 mg, followed by 75 mg per day) in patients with ACS. The primary composite outcome of cardiovascular deaths, nonfatal myocardial infarction, or stroke was 20% lower in the clopidogrel group (9.6% vs. 11.4%). This was mainly driven by a 23% reduction in recurrent myocardial infarction (MI). Compared to placebo, DAPT with clopidogrel increased the risk of major bleedings (requiring the transfusion of two or more units of blood) by 38% (gastrointestinal and at the sites of the arterial puncture), and need for transfusion by 30%, with no excess of fatal or life-threatening bleeding [30].

**Figure 2:**
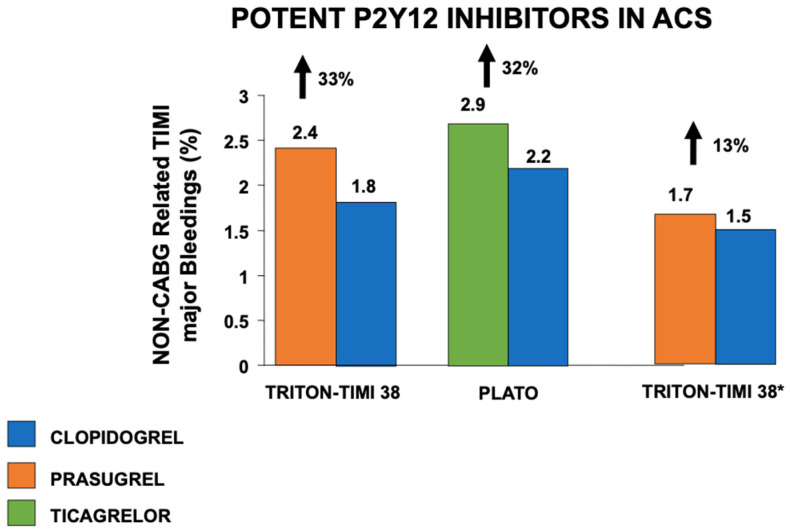
Impact of potent vs. non-potent P2Y12 inhibitors in association with aspirin on non-CABG-related TIMI major bleeding complications in patients with acute coronary syndromes. *Analysis restricted to patients < 75 years, without previous stroke, weight > 60 kg. ACS—Acute Coronary Syndrome; CABG—Coronary Artery Bypass Graft; TIMI—Thrombolysis in Myocardial Infarction.

Additional large trials conducted in the setting of ACS have shown the superiority of both prasugrel (TRITON-TIMI 38 trial, among those undergoing invasive treatment) [31] and ticagrelor (PLATO trial) [32] as compared to clopidogrel in ischemic endpoints, despite a higher risk of bleedings (Figure 2).

Currently approved oral P2Y12 inhibitors could be classified based on the pharmacological class into thienopyridines (clopidogrel and prasugrel) and non-thienopyridines (ticagrelor). A more clinical classification is based on the potency and consistency of platelet inhibition in potent (prasugrel, ticagrelor) and non-potent (clopidogrel) agents [33].

In brief, the current choice of P2Y12 inhibitor for DAPT is mainly based on the clinical presentation: current guidelines recommend using a potent P2Y12 inhibitor (prasugrel or ticagrelor) on top of aspirin in patients undergoing PCI for ACS if there are no contraindications or the patient does not have a high bleeding risk [34]. As the efficacy of clopidogrel is hampered by the slow and variable transformation of the prodrug into active metabolites, the highest thrombotic risk associated with an acute presentation requires a more intense antithrombotic treatment [35]. In turn, in a patient with chronic coronary syndrome (CCS), clopidogrel is the P2Y12 inhibitor of choice, unless additional high ischemic risk characteristics are present. 

## 3. Optimal DAPT Duration

### 3.1. Twelve Months DAPT

In the last ten years, there has been a great number of studies exploring optimal duration of DAPT followed by a single antiplatelet agent. Initially the recommended duration for DAPT after coronary stenting was 1–6 months [36]. This treatment window was non-randomly tested in the studies that compared drug-eluting stents (DES) with bare metal stents (BMS). The mean follow-up time for clopidogrel-based DAPT in the PCI CURE trial was 8 months [16]. The evidence of an increased risk of late and very late ST after first-generation DES led to a more cautious approach with extension of DAPT well beyond 12 months after implantation [37,38,39,40]. Based on the available evidence, consensus-based guidelines recommended a minimum of 12 months of DAPT in this setting [41]. However, the introduction of safer stent platforms [42,43] progressively showed that DAPT duration could be safely shortened [44].

The main reason to strike an optimal duration of DAPT is to reasonably balance the risk of ischemic and bleeding events, both impacting prognosis, and both opposingly affected by the duration of antithrombotic therapy. In this context, trials comparing three or six months of DAPT to one-year DAPT found no difference in ischemic events and showed that a short-term DAPT was safer in terms of bleeding [45]. 

For patients presenting with CCS, 2019 ESC guidelines for the management of chronic coronary syndromes recommend a 6-month DAPT (ASA and clopidogrel) regimen after PCI (Class I, level A). Prasugrel or ticagrelor may be considered in high-risk situations, such as suboptimal stent deployment or complex PCI [46].

In patients with ACS, 2023 ESC guidelines for the management of ACS recommend a default strategy of 12 months of DAPT with a potent P2Y12 inhibitor (Class I); however, if the patient undergoes PCI, prasugrel should be considered as the P2Y12 inhibitor of choice, considering the results of the ISAR-REACT 5 RCT. Clopidgrel should only be used when ticagrelor and prasugrel are contraindicated, not available or in HBR patients [34]. 

### 3.2. Prolonged (>12 Months) DAPT

Compared to bare-metal stents, DESs reduced the rate of restenosis; however, there was an initial concern that first-generation DESs may be associated with an additional risk of late and very-late ST [47]. In addition, beyond stent-related events, ischemic events unrelated to the treated plaque may also occur, which supports the potential for prolonged antiplatelet therapy as a secondary prevention strategy [48,49].

Patients with MI have heightened platelet activation and aggregation compared with patients with CCS, leading to a higher predisposition to atherothrombosis [50,51,52], which may persist for years afterwards [53,54,55]. Hence, these patients may benefit from more intensive antiplatelet therapies following PCI.

The DAPT study was a multicenter randomized trial that enrolled patients treated with DAPT after PCI with DES. At 12 months, patients who had no MACCE, another revascularization, bleedings (moderate to severe) and had been adherent to the therapy were randomized to continue it or to placebo for another 18 months, to compare the 12 month-strategy to the 30-month strategy. The efficacy end points (cumulative incidence of definite or probable ST and the composite of death, MI, or stroke) were significantly lower in the group that continued thienopyridine (0.4% vs. 1.4% for stent thrombosis; 4.3% vs. 5.9% for MACCE) and was consistent across stent type and thienopyridine drug used. However, prolonged DAPT led to a significantly higher rate of bleeding, and all-cause mortality was increased by 36% in the prolonged DAPT group [56]. Importantly, most of the benefit observed with a prolonged DAPT treatment was observed among patients presenting with a MI [57,58].

Ticagrelor, when added to aspirin after an ACS, compared with clopidogrel, reduces the rate of major adverse CV events. Therefore, patients that suffered a MI, who are at higher risk for recurrent ischemic events, could benefit from prolonged DAPT with ticagrelor. 

The Prevention of Cardiovascular Events in Patients with Prior Heart Attack Using Ticagrelor Compared to Placebo on a Background of Aspirin–Thrombolysis in Myocardial Infarction 54 (PEGASUS-TIMI 54) trial, a randomized, double-blind, placebo-controlled clinical trial, tested long-term DAPT with ticagrelor, evaluating two different doses: 90 mg twice daily and 60 mg twice daily. 

The patients were considered eligible if they had a spontaneous MI 1 to 3 years before, were 50 years old or older, and had one of these additional high-risk features: age of 65 years or older, diabetes mellitus (DM), a second prior spontaneous MI, multivessel coronary artery disease, or chronic renal dysfunction. They were randomly assigned in a 1:1:1 ratio to receive either ticagrelor at a dose of 90 mg twice daily (b.i.d.), ticagrelor at a dose of 60 mg b.i.d., or a placebo. The median time from the occurrence of MI, of which 53.6% were STEMI, to the point of randomization was 1.7 years. Both doses of ticagrelor reduced the incidence of cardiovascular death, MI, or stroke but led to a higher risk of bleeding, including TIMI major bleeding, bleeding requiring a transfusion, and bleeding leading to discontinuation of the study drug. Ultimately, the overall risk to benefit trade-off was improved with the ticagrelor 60 mg b.i.d. dose, which led to the final approval of this drug dose with the new indication for extended DAPT [59]. 

Udell et al. conducted a meta-analysis of six RCTs on secondary prevention, including 33,435 patients with prior-MI randomized to extended DAPT beyond 1 year or standard DAPT for 12 months. A DAPT regimen longer than 12 months led to a 22% reduction in the relative risk and a 1.1% reduction in the absolute risk of MACE during an average follow-up period of 31 months. There was a slight 0.8% absolute increase in the risk of major bleeding, but this did not result in a significant excess of ICH or fatal bleeding, and there were no significant differences in non-cardiovascular deaths. Importantly, extended DAPT was associated with a significant reduction in CV mortality [60]. Taking this into consideration, extended DAPT appears to be an attractive approach in patients with prior MI and a low risk for bleeding. Whether the type of P2Y12i may be associated with a different impact on the overall long-term outcomes is not well established [61].

According to current ESC guidelines, a prolonged DAPT duration should be considered in patients with high ischemic risk and without HBR (Class IIa) and may be considered in patients with moderate ischemic risk without HBR (Class IIb) [34].

### 3.3. Short (1 or 3 or 6-Months) DAPT

A short DAPT strategy of less than 12 months after implanting a coronary stent has been compared to one-year DAPT in several RCTs, generally testing non-inferiority for ischemic events and superiority for bleeding. 

The EXCELLENT trial (A Comparison of Xience/Promus Versus Cypher in Reducing Late Loss After Stenting) aimed to assess the effectiveness of a short-term DAPT strategy (6 months). A total of 1443 patients were randomized according to DAPT duration (6 vs. 12 months) and the type of stent (everolimus-eluting stent vs. sirolimus-eluting stent). One of the points of the study was to determine if the short-term DAPT strategy was not inferior to the standard care concerning the occurrence of cardiac death, MI, or ischemia-driven target vessel revascularization (TVR). 

The primary endpoint of the study was in-stent late loss at 9 months as the primary endpoint when comparing stent types, while the co-primary endpoint was the occurrence of target vessel failure (TVF) at 12 months, in relation to the duration of DAPT. The findings revealed that the six-month DAPT strategy was as effective as the standard care. Furthermore, while there was a numerical rise in TIMI minor and major bleeding events in the one-year DAPT group, this difference did not reach statistical significance (HR 0.40; 95% CI: 0.13–1.27; *p* = 0.12) [62]. 

The PRODIGY (PROlonging Dual antIplatelet treatment after Grading stent-induced intimal hYperplasia study) trial was a 4:2 randomized open-label clinical trial that evaluated the efficacy and the safety of prolonged DAPT with clopidogrel as P2Y12 inhibitor, randomly allocating patients (predominantly presenting with ACS) to four coronary stents (BMS, paclitaxel-eluting stent, E-ZES, everolimus-eluting stent) and two DAPT duration regimens (6 vs. 24 months of DAPT). The study showed no differences in the primary efficacy endpoint (death, MI, and stroke between 6 and 24 months of DAPT), and, as expected, among the patients receiving 24-month DAPT, there was a risk of type 2, 3 or bleeding events two-fold greater than in the group receiving 6-month DAPT (HR 2.17, 95% CI 1.44–3.22; *p* = 0.00018) [63]. Results remained largely consistent in multiple subgroups [64,65,66].

The REDUCE trial randomized 1496 ACS patients treated with COMBO stent to 3 or 12 months of DAPT. The composite primary study endpoint of all-cause mortality, MI, ST, stroke, target vessel revascularization and bleeding at 12 months was similar in the two groups, reaching prespecified non-inferiority (8.2% vs. 8.4%, *p*-value non-inferiority < 0.001). However, numerically higher rates of mortality and ST in the three-month DAPT group were observed [67]. The non-inferiority of 3 vs. 12 months DAPT was confirmed in several high-risk subgroups [68,69,70].

After 3–6 months of DAPT, in patients who are event-free and who are not at high ischemic risk, SAPT with a P2Y12 inhibitor or aspirin should be considered according to the current ESC guidelines (Class IIa) [34]. 

### 3.4. Individualization of DAPT Duration Based on Ischemic and Bleeding Risk

**Figure 3:**
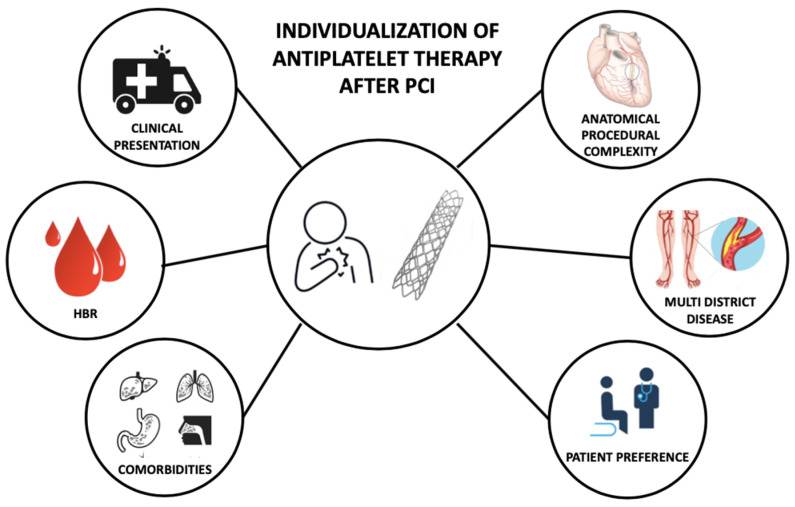
Features informing individualization of antiplatelet therapy after percutaneous coronary intervention. PCI—Percutaneous Coronary Intervention; HBR—High Bleeding Risk.

The optimal DAPT strategy, that maximizes the efficacy and the safety of the treatment, balancing ischemic and bleeding risk, should be individual and selected based on patient and procedural characteristics (Figure 3) [71,72,73].

The clinical presentation (ACS vs. CCS) at the time of PCI is an important feature that influences the patient’s mortality risk (ranging between 0.36% in CCS and 4.78% in high-risk STEMI patients), and the risk of recurrent ischemic events [74,75]. Clinical presentation in the setting of an all-comer PCI population is also a treatment modifier of DAPT duration after coronary stenting. Costa et al. conducted an analysis of the PRODIGY trial, a study designed to compare 6- or 24-month DAPT regimens in patients treated with PCI with first- and second-generation DES with varying anti-intimal hyperplasia potency. It showed the lack of ischemic benefit in favor of a 2-year course of DAPT, in both CCS and ACS patients. In both CCS and ACS patients, the rate of bleeding was higher in the 24-month DAPT arm, with a greater magnitude in patients presenting with CCS. In terms of NACE rates, after excluding BARC 2 bleeding, there was a numerical increase in NACE in CCS patients only, with consistent borderline results in the 24-month DAPT arm, provided by the interaction testing. So, this analysis showed that clinical presentation plays a pivotal role in the treatment decision with respect to the value of DAPT duration (prolonged vs. shortened) [66].

In the DAPT trial, a randomized double-blind, placebo-controlled trial comparing 30 versus 12 months of DAPT after stent implantation, 30-month DAPT compared with one-year treatment reduced definite or probable ST in patients with MI (0.5 vs. 1.9%; *p* = 0.001) and without MI (0.4 vs. 1.1%; *p* = 0.001) (Pint = 0.69). The reduction in MACCE was greater for patients with MI (3.9 vs. 6.8%; *p* = 0.001) than patients without MI (4.4 vs. 5.3%; *p* = 0.08) at the time of presentation. Of 11,648 randomized patients (9961 treated with drug-eluting, 1687 with bare metal stents), 3576 (30.7%) presented with MI. Among patients presenting without MI, a longer DAPT regimen was also associated with a significant increase in all-cause death (2.1 vs. 1.5%; *p* = 0.04) [56]. 

The risk of ischemic events following PCI is also influenced by anatomical and procedural factors. These factors have consistently been recognized as crucial considerations when determining the duration of DAPT [76,77]. The complexity of a PCI can be quantified using previously validated and guideline-endorsed criteria: PCI with ≥3 stents implanted and 3 ≥ lesions and/or coronary vessels treated; and/or bifurcation with 2 stents implanted, total stent length > 60 mm, and/or treatment of a chronic total occlusion (CTO). In these patients, long-term DAPT (≥12 months) compared with a short period of DAPT (3 or 6 months), significantly reduced the risk of cardiac ischemic events [78].

Even the localization of the coronary artery stenosis should be considered a treatment modifier for DAPT duration, as suggested by Costa et al. in a retrospective analysis of the PRODIGY trial. In patients with a stenosis of at least 30% on angiography, appraised by visual estimation, of the left main or the proximal LAD, a 24-month DAPT regimen, compared to a 6-month DAPT regimen, significantly reduced the rate of definite, probable, or possible ST. A consistent interaction between CAD location and DAPT duration was also registered for the composite of CV death and MI. The 2-year DAPT regimen remained associated with possible benefits in patients with LM/pLAD lumen narrowing irrespective of stent implantation in these segments or patients’ clinical presentation [79].

In a patient-level meta-analysis comprising data from six RCTs (with a total of 9577 patients) that examined the optimal duration of DAPT following coronary stenting (comparing 12 months to 6 months), 17.5% of the patients exhibited characteristics associated with complex PCI. These characteristics included three-vessel PCI, implantation of three or more coronary stents, treatment of three or more coronary lesions, bifurcation stenting (using stents in both the main and side branches), a final total stent length exceeding 60 mm, and treatment of a chronic total occlusion (CTO). In this group, long DAPT compared with short DAPT reduced the adjusted MACCE rate (unadjusted event rates: 4.0 vs. 6.0%; adjusted HR 0.56, 95% CI 0.35–0.89), whereas, in the non-complex PCI group, no benefit for a longer treatment was observed (2.5 vs. 2.6%; adjusted HR 1.01; 95% CI 0.75–1.35) (Pint = 0.01). The magnitude of benefit in favor of long DAPT was directly related to the complexity of the procedure [80].

Concomitant high bleeding risk can mitigate the benefit from a longer DAPT regimen in patients undergoing a complex PCI. In an analysis that included 14,963 patients from eight randomized trials, long-term DAPT in non-HBR patients reduced the ischemic events in both complex and noncomplex PCI, but not among HBR (PRECISE-DAPT > 25) patients, regardless of complex PCI features [81].

Age is a relevant clinical factor to appraise in the choice of DAPT regimen. Although older patients (75 years old or older), have an increased thrombotic risk [82], age also represent a minor bleeding risk factor; indeed, it is included as one of the minor HBR criteria, due likely owing to concomitant renal impairment or anemia [83]. Common general strategies to reduce the rate of bleeding in this group of patients are use of proton pump inhibitors, use of radial arterial access for coronary angiography and PCI and a modulation of DAPT composition and duration [83]. According to current ESC guidelines, clopidogrel is the preferable P2Y12 inhibitor, due to a better safety profile. However, in elderly patients with a high thrombotic risk, the DAPT regimen has to be tailored, taking into account that, in the first months after the index event, the thrombotic risk is higher; therefore, a de-escalation strategy seems reasonable [84].

Gender-related differences have not been shown to affect the efficacy and safety of DAPT type or duration. No heterogeneous findings across genders have been found in pooled analysis or trials assessing a 12-year DAPT or longer vs. a shorter one [85]. A borderline quantitative interaction between reduction in ST and prolonged DAPT suggesting a relative treatment benefit in female patients compared to male patients has been shown in the DAPT trial; however, no differences were shown for MACCE or bleeding [56]. 

Current ESC guidelines recommend a similar type and duration of DAPT in male and female patients (Class I) [85]. 

Diabetes mellitus (DM) represents a risk modifier in patients presenting with both CCS and ACS, increasing the risk of fatal and non-fatal ischemic events. In terms of P2Y12 inhibitor type, there is no evidence that DM should affect the decision; indeed, no difference in benefits was shown in the CURE, the TRITON-TIMI 38 or the PLATO trial in patients with DM as compared to those without DM [16,31,32]. The DAPT study showed a lower risk reduction for MI in patients with DM but no differences for all other ischemic and safety endpoints [86]. The PEGASUS trial showed no difference for primary efficacy endpoint with respect to the presence or absence of DM [87]. Therefore, even in terms of DAPT duration, DM should not be the only patient feature to be taken into consideration, and similar DAPT type and duration should be considered in both patients with and without DM (Class IIa) [85].

Patients with peripheral artery disease are at high ischemic risk. In the CHARISMA trial, in 3096 patients, DAPT (clopidogrel plus aspirin), compared to aspirin alone, reduced the rate of MI, with no differences in terms of moderate, severe, or fatal bleeding. However, there was an increase in minor bleeding [88]. In a subgroup analysis of the PEGASUS study, the investigators found that patients with PAD and prior MI had a 60% higher risk of MACE compared to patients without PAD. In this group of patients, ticagrelor 60 mg b.i.d., compared to placebo, granted an absolute risk reduction in ischemic events of 5.2% at 3 years [89]. In the PRODIGY trial, PAD was associated with a higher risk of death and ischemic events, and prolonged DAPT, compared to short DAPT, reduced the primary efficacy endpoint in patients with PAD [90]. The ESC guidelines, considering this evidence, suggest that a prolonged DAPT (>12 months) may be considered in patients with PAD (Class IIb) [85].

### 3.5. Optimal DAPT Duration in HBR Patients

The optimal duration of DAPT in high-bleeding-risk patients (HBR) has been recently explored. DAPT leads to a significant increase in the rate of major bleeding, associated with greater risks of adverse cardiac outcomes. Major bleeding is associated with an immediate and sustained increased risk of mortality, similar or greater than recurrent MI, easily offsetting the benefit of its ischemic protection. Roughly one out of three patients undergoing PCI is at HBR (i.e., patients of older age, low hemoglobin level, thrombocytopenia, renal insufficiency, cancer, a prior stroke, and a bleeding history) [24]. HBR features are also associated with an increased ischemic risk. These patients are considered to be at double-sided risk: high risk for bleeding, and high risk for recurrent ischemic or thrombotic events. This is why weighing in ischemic and bleeding risks to decide the optimal DAPT strategy is a challenge for the clinician [91]. 

The current guidelines endorse the use of a standardized tool, the Predicting bleeding complications in patients undergoing stent implantation and subsequent DAPT therapy (PRECISE-DAPT) score for bleeding risk stratification [85]. The PRECISE-DAPT score was developed to predict the bleeding risk in patients undergoing PCI and, therefore, a DAPT regimen. This score is based on five features of clinical and laboratory features: age, white blood cell count, hemoglobin level, creatinine clearance, and history of spontaneous bleeding. Patients with a score of ≥25 are at a high risk of bleeding, indicating that the DAPT duration should be shorter (3 to 6 months) than that in patients with a score of <25. To derive and validate the score, Costa et al., pooled 14,963 patients treated with DAPT after coronary stenting from eight multicenter randomized clinical trials. Using Cox proportional hazards regression, predictors of TIMI major or minor bleeding were identified, and based on them, a numerical score was developed. A derivation cohort of patients was used to assess the predictive performance of the score, and it was validated in validation cohorts of patients from the PLATO trial (n = 8595) and BernPCI registry (n = 6172). In patients identified at high risk (score ≥ 25), a longer DAPT duration significantly increased bleeding, but not in those with lower risk profiles [92]. Multiple external validations of this prediction tool have been presented, largely confirming the score discriminative ability [93,94].

A simplified version of the score, the four-item PRECISE-DAPT, lacking white blood cells count, has been studied for its possible potential to guide DAPT. In an analysis carried out in a pooled dataset of five randomized studies and including more than 10,000 patients, it showed that it may be useful to support clinical decision making for DAPT duration [95]. 

The Academic Research Consortium for High Bleeding Risk (ARC-HBR), a collaboration of leading research organizations, regulatory authorities, and physician-scientists from the United States, Asia, and Europe focused on PCI–related bleeding, elaborated a standardized definition of HBR patients, based on a review of the available evidence. Twenty clinical criteria were identified as major or minor by the consensus. Patients are considered to be at HBR if at least one major or two minor criteria are met. The coexistence of numerous numbers of risk factors is associated with a linear increase in risk of BARC 3 to 5 bleeding. Multiple external validations of this classification have been recently published [96].

The optimal DAPT duration for limiting bleeding risk while not reducing ischemic protection was investigated in the MASTER-DAPT trial. MASTER-DAPT was the first randomized trial to demonstrate that among HBR patients undergoing PCI with a bioresorbable polymer-based sirolimus-eluting stent, a 1-month DAPT regimen was non-inferior to a standard DAPT in terms of NACE (a composite of death from any cause, myocardial infarction, stroke, or major bleeding), or MACCE (a composite of death from any cause, myocardial infarction, or stroke), and superior in terms of major or clinically relevant non-major bleeding (MCRB). The trial enrolled patients, regardless of their clinical presentation, that had undergone successful PCI with implantation of a biodegradable polymer sirolimus-eluting stent (Ultimaster, Terumo), at HBR. Patients had to be free from ischemic and active bleeding events at the moment of the randomization. They were randomly assigned to an abbreviated DAPT regimen (i.e., stopped DAPT after 30 days and continued SAPT; abbreviated-therapy group) or to standard DAPT regimen (i.e., 6 months after the index procedure; standard-therapy group). The aim of the trial was to test whether the abbreviated dual antiplatelet therapy was non-inferior in terms of net adverse clinical events (NACE), major adverse cardiac or cerebral events (MACCE), and superior with regard to major or clinically relevant bleeding, compared to standard regimen. The trial showed that in patients at high risk for bleeding, the discontinuation of DAPT at a median of 34 days after PCI led to a reduction in the incidence of major or clinically relevant non-major bleeding (6.5% in the abbreviated-therapy group and 9.4% in the standard-therapy group) and was non-inferior with regard to NACE and MACCE compared to standard treatment [97].

The XIENCE 28 and XIENCE 90 studies were two prospective, single-arm studies, that compared a 1-month DAPT regimen (XIENCE 28) and a 3-month DAPT regimen (XIENCE 90), in HBR patients that underwent successful PCI with a Xience stent implanted, a durable polymer everolimus-eluting stent, with a 12-month DAPT, testing the hypothesis that a short DAPT regimen would be non-inferior in terms of ischemic events and superior with respect to bleeding events. A total of 3652 patients, free from MI, repeat coronary revascularization, stroke, or ST, were randomized to 1-month (1605) or 3-month (2047) DAPT. Patients that presented with STEMI or with a left ventricular ejection fraction <30% were excluded. Most of the patients continued with ASA after DAPT discontinuation. The primary study endpoint was all-cause death or all MI; the secondary endpoints were BARC 2–5 bleeding and definite/probable ST. The studies present limitations inherent to the non-randomized design. A short DAPT regimen lasting either 1 or 3 months demonstrated non-inferiority concerning ischemic events. It also exhibited comparable rates of clinically relevant bleeding events (BARC 2–5), along with a notable decrease in major bleeding (BARC 3–5). Importantly, there was a remarkably low occurrence of stent thrombosis [98,99].

**Figure 4:**
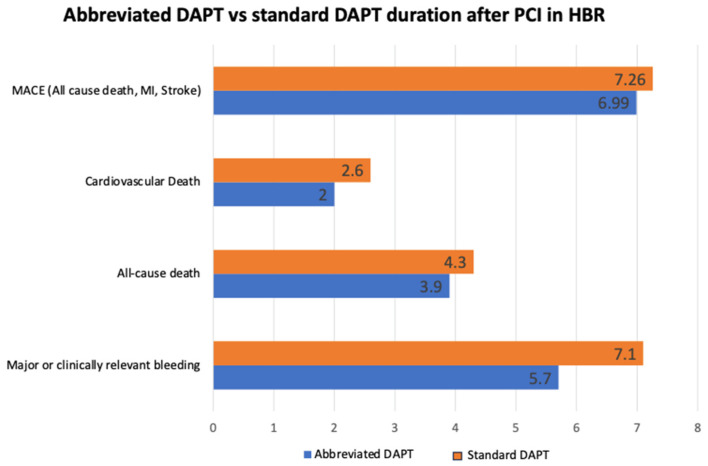
Impact of abbreviated vs. standard dual antiplatelet therapy duration on clinical events in patients at high bleeding risk. DAPT—Dual Antiplatelet Therapy; PCI—Percutaneous Coronary Intervention; HBR—High Bleeding Risk; MI—Myocardial Infarction; MACE—Major Adverse Cardiovascular Events.

A recent metanalysis of RCTs searched studies comparing abbreviated (1-month) or short (3-month) with standard (≥6-month) DAPT in HBR patients (PRECISE-DAPT > 25) with no indication for anticoagulant therapy to estimate the impact of these abbreviated DAPT regimens in this patient setting. Articles were initially screened by title and abstract content. A total of 11 RCTs, including 9006 patients that underwent PCI who were randomized to abbreviated (≤3 month) or standard (≥6 month) DAPT regimens, and reporting bleeding and ischemic endpoints at a minimum follow-up of 12 months after enrollment, were included. A lower rate of major or clinically relevant non-major bleeding, major bleeding and CV mortality was registered with the abbreviated DAPT regimens, with no significant differences in terms of prevention of MACE, ST, and other ischemic events, irrespective of clinical presentation and P2Y12i used (Figure 4) [100].

The risk factors for both bleeding and ischemic events can intersect, leading to an ongoing debate regarding the most effective approach to reduce both types of events in this particular patient population. The OPT-BIRISK trial is a multicenter, double-blind, placebo-controlled superiority trial comparing clopidogrel monotherapy with DAPT, aspirin and clopidogrel, in patients with both high bleeding and ischemic risk who have completed 9–12 months of DAPT after PCI for ACS. The primary endpoint of BARC 2, 3 or 5 bleeding and the key secondary endpoint of MACCE (a composite of all-cause mortality, MI, stroke or clinically driven revascularization) were lower in the clopidogrel monotherapy arm, 2.5% vs. 3.3%, (HR 0.75, 95% CI 0.57–0.97, *p* = 0.03) and 2.6%. vs. 3.5%, (HR 0.74, 95% CI 0.57–0.96, *p* = 0.02). The majority of patients enrolled in the study presented with unstable angina, which could potentially influence the observed outcomes. However, it is important to consider that patients were not randomized into treatment groups until 9–12 months after undergoing PCI. This timing suggests that the patient population was transitioning toward the chronic phase within the spectrum of ACS/CCS [101].

One-month DAPT, followed by aspirin or P2Y12 receptor inhibitor monotherapy, may be considered in HBR patients, according to current ESC guidelines (Class IIb) [34].

### 3.6. Patients Treated on Long-Term OAC

The need for the association of antiplatelet and anticoagulant therapies is frequent, mostly in patients affected by atrial fibrillation who need thromboembolic prevention (AF), undergoing PCI or with concomitant ACS (a condition that occurs in about 10% of cases of hospital admission for ACS) [102]. Anticoagulant therapy is inferior to antiplatelet therapy after stent implantation to prevent recurrent ischemic events and ST; on the other hand, antiplatelet therapy is inferior to anticoagulant therapy to prevent stroke and systemic embolism in patients with AF [103]. However, triple therapy with oral anticoagulation and dual antiplatelet therapy (TAT) exposes the patient to an excessive bleeding hazard (3–4 times higher than the two treatments considered alone) [104]. Hence, the patient needing long-term oral anticoagulation is considered HBR per se. 

Considering that long-term OAC therapy is mandatory in this setting, reducing DAPT duration has been widely explored in multiple clinical trials, testing the hypothesis of removing one antiplatelet agent early after stenting, downgrading TAT to a dual antithrombotic therapy (DAT) [105]. 

Several studies focused attention on this clinical setting, studying the duration and the intensity of the antithrombotic treatment. 

Multiple trials evaluated, in patients with AF undergoing PCI, the safety and efficacy of a DAT therapy approach with a NOAC compared with a triple antithrombotic therapy with a vitamin K antagonist [106,107].

The PIONEER AF-PCI trial randomized 2124 patients affected by non-valvular AF treated with PCI to receive DAT consisting of low-dose rivaroxaban (15 mg die) plus a P2Y12 inhibitor; very low-dose rivaroxaban (2.5 mg die) plus DAPT, or a VKA plus DAPT. At 12 months, the study showed a lower rate of relevant bleeding in the groups not receiving a VKA, with the lowest rate in the low-dose rivaroxaban group (6.8% for rivaroxaban 15 mg + P2Y12i, 18.0% for rivaroxaban 2.5 mg + 26.7% for a VKA + DAPT). Rates of ischemic events did not significantly differ in the three groups [106].

The AUGUSTUS trial, a randomized trial with a 2:2 factorial design, enrolled 4614 patients with AF who had an ACS or had undergone PCI and were planning to take a P2Y12 inhibitor and randomized them to receive apixaban or a vitamin K antagonist and to receive aspirin or placebo for 6 months. AUGUSTUS tested OAC type and dual vs. triple therapy separately. At six-month follow-up, the primary endpoint, the rate of MCRB, was 10.5% of the patients receiving apixaban, as compared with 14.7% of those receiving a VKA. With respect to the second randomization, the primary endpoint occurred in 16.1% in the aspirin group, as compared to 9.0% in the placebo group (HR, 1.89; 95% CI, 1.59–2.24; *p* < 0.001). With respect to ischemic events, the incidence rate was consistent among patients who received apixaban or a vitamin K antagonist (VKA), as well as among those randomly assigned to aspirin or a placebo. Notably, the apixaban group experienced a 50% reduction in ischemic strokes. In conclusion, in patients with AF and a recent ACS or PCI, both dual therapy and apixaban were associated with a reduction in bleeding without an increase in ischemic events [108]. 

The impact of dual or triple therapy in HBR patients undergoing PCI has been evaluated in a subgroup analysis of the REDUAL PCI trial. In this study, DAT with dabigatran 110 mg b.i.d. reduced bleeding risk in both patients at HBR and non-HBR. In turn, DAT with dabigatran 150 mg, compared to warfarin-based TAT, reduced bleeding in non-HBR patients but not in HBR patients, with a trend towards less benefit in HBR patients [109]. 

Importantly, while most of the trials tested a DAT approach linked to NOAC and a TAT linked to a VKA [110,111], it is important to unravel the true benefit associated with dual therapy beyond those given by a treatment with NOAC, which per se is associated with less bleeding compared to a VKA. On this matter, Montalto et al. performed a systematic review evaluating randomized controlled trials comparing an abbreviated (4–6 weeks) or prolonged (≥3 months) DAPT regimen in patients with OAC. Co-primary endpoints were MCRB and major bleeding, while the rate of MACE was the endpoint for safety. Five studies were included, for a total of 7665 patients, 3843 treated with abbreviated DAPT and 3822 with prolonged DAPT. MCRB and major bleeding were lower with abbreviated DAPT [risk ratio (RR) 0.69 (0.52–0.91); *p* = 0.01 and 0.70 (0.52–0.95); *p* = 0.01, respectively] while the rate of MACE and other ischemic events (CV death, ST, MI) did not differ. Network meta-analysis showed that peri-procedural DAPT had the highest probability of being the best treatment in preventing MCRB and major bleeding when compared with both short and longer DAPT strategies [112]. 

Current ESC guidelines recommend, for patients with AF suffering an ACS and undergoing PCI, a strategy of TAT, with clopidogrel as P2Y12 receptor inhibitor, for up to 1 week, then DAT (NOAC and preferably clopidogrel) for up 12 months (Class I). When using rivaroxaban or dabigatran as NOAC in HBR patients, a lower dose (15 mg o.d. for rivaroxaban and 110 mg b.i.d. for dabigatran) should be considered to mitigate the bleeding risk (Class IIa). TAT for longer than 1 week and up to 1 month should be considered in patients with high ischemic risk if it outweighs the bleeding risk (Class IIa). Withdrawing antiplatelet therapy at 6 months while continuing OAC may be considered (Class IIb) [34]. 

## 4. Options for DAPT Discontinuation

After an initial period with DAPT, discontinuation of this regimen could be either followed by aspirin or P2Y12 inhibitor monotherapy. Multiple RCTs evaluated if a shorter-term DAPT strategy for 6, 3 or even 1 months after DES implantation, followed by ASA monotherapy, was non-inferior to 12 months of DAPT for ischemic endpoints while providing a reduction in bleeding events. More recently, a short DAPT strategy for 1–3 months followed by P2Y12 inhibitor monotherapy vs. standard DAPT was tested in multiple RCTs. Most of the patients in the P2Y12-inhibitor monotherapy arm of these trials were treated with ticagrelor. In a systematic review and individual participant data meta-analysis, Valgimigli et al. evaluated all randomized trials that compared P2Y12 inhibitor monotherapy after DAPT with DAPT among patients who underwent coronary revascularization. Among the 24,096 patients included, P2Y12i monotherapy was non-inferior to longer-term DAPT for the composite endpoint of all-cause death, MI, and stroke throughout (2.95% vs. 3.27; *p* = 0.005 for non-inferiority). This strategy also reduced the risk of BARC type 3 or type 5 bleeding among patients randomly allocated to P2Y12 inhibitor monotherapy [113]. Interestingly, these results were consistent also when higher-risk patients treated with complex PCI were evaluated [114].

### 4.1. ASA or P2Y12 Inhibitors for Long-Term Single Antiplatelet Therapy

While international guidelines endorse long-term treatment with aspirin in patients with prior ischemic events [115], which in practice often translate to a lifetime commitment to this therapy, whether a P2Y12 inhibitor might represent a better option to the aspirin paradigm has been a matter of debate. 

In the CAPRIE trial, which randomized patients with a recent ACS to clopidogrel vs. aspirin monotherapy, clopidogrel was associated with a small but statistically significant 8.7% relative reduction in the primary ischemic endpoint compared to aspirin, with a favorable safety profile with fewer hemorrhagic events, especially gastrointestinal bleedings [116].

In a large systemic review and study level meta-analysis including 42,108 patients, Chiarito et al. observed that P2Y12 inhibitor monotherapy is associated with a modest significant reduction in the risk of MI compared with those who received aspirin (OR 0.81 [95% CI 0.66–0.99]; I2 = 10.9%) while the risk of stroke, all-cause death, vascular death and major bleeding did not differ between the two groups [117].

The HOST-EXAM trial (Harmonizing Optimal Strategy for Treatment of Coronary Artery Stenosis–Extended Antiplatelet Monotherapy) randomized patients who underwent uneventful DAPT for 12 ± 6 months after PCI to discontinue DAPT, continuing with either aspirin or clopidogrel. The study demonstrated that clopidogrel monotherapy was superior to aspirin monotherapy during the chronic maintenance period with regard to net adverse clinical events (5.7% vs. 7.7%, HR 0.73, 95% CI: 0.59–0.90, *p* < 0.01) during the 24-month follow-up. The study’s extended follow-up also showed that clopidogrel monotherapy was associated with a 26% risk reduction in the primary end point, similar to the results during the in-trial period, with no difference in terms of all-cause death between aspirin and clopidogrel [118]. This randomized comparison importantly challenges the central role of aspirin in the long-term management of vascular disease patients.

Consistently, in a recent patient-level meta-analysis of seven randomized trials and 24,325 patients, Gragnano et al. compared P2Y12 inhibitor vs. aspirin monotherapy in patients with established CAD. The rate of the primary outcome, a composite of CV death, MI, and stroke, was lower with P2Y12 inhibitor monotherapy compared with aspirin over a 2-year follow-up, mainly driven by a significant reduction in MI (HR: 0.77; 95% CI: 0.66–0.90; *p* < 0.001), with similar rates of major bleeding and a more favorable net adverse clinical events profile in the P2Y12i group [119]. The SHARE (SHort-term Dual Antiplatelet Therapy After Deployment of BioabsoRbable Polymer Everolimus-Eluting Stent) trial was a study with the aim to compare the efficacy and safety of P2Y12 inhibitor monotherapy after 3 months of DAPT vs. a 12-month DAPT in patients treated with DES implantation. The P2Y12i used were clopidogrel for patients with CCS and ticagrelor for patients with ACS. The study met the non-inferiority criteria for the primary outcome of NACE (a composite of MACCE and major bleeding) at 3 months, −0.44% (95% CI, −1.19 to 0.32) *p* for non-inferiority < 0.001, and 12 months, −0.93% (95% CI, −2.64 to 0.77) *p* for non-inferiority < 0.001. The P2Y12i monotherapy also reduced the rate of major bleeding (0.2% vs. 0.8%) [120]. 

The GLOBAL LEADERS trial aimed at comparing 1 month of DAPT with ticagrelor followed by 23 months of ticagrelor monotherapy with 12 months of DAPT (aspirin plus clopidogrel or ticagrelor) followed by aspirin monotherapy in patients treated with PCI with a biolimus-eluting stent. The experimental strategy was non-inferior but not superior to the standard strategy. The primary outcome (a composite of all-cause mortality and nonfatal MI) occurred in 3.8% in the ticagrelor monotherapy group compared with 4.4% in the control group, and the rate of grade 3 or 5 bleeding was similar (2.5% vs. 2.5%) [121].

### 4.2. Immediate DAPT Discontinuation after PCI, the No DAPT Strategy

With a trend towards exploring the feasibility of a progressively shorter DAPT after PCI with 6-, 3- and even 1-month periods, the complete omission of aspirin immediately after PCI appears as a natural extension of the short-DAPT strategy. This concept has been first studied in the ASET (Acetyl Salicylic Elimination Trial), a multicenter pilot study, of an aspirin-free prasugrel monotherapy strategy tested immediately after PCI in patients presenting with CCS undergoing non-complex PCI. The exploratory study design, based on a stop rule in the event that more than three coronary ST were observed, showed that this strategy was feasible with no ST observed, while ischemic events were rare [122]. 

This strategy was also recently studied in ACS patients in the OPTICA and MACT trial, which resembled the study design of the ASET [123,124]. In this latter trial, which was also the only one to include STEMI patients, two ST were observed, all in this higher risk group, which raises concerns regarding early de-escalation of treatment in patients at particularly high ischemic risk such as STEMI patients [123,125].

The recent STOPDAPT-3 trial compared an aspirin-free strategy with prasugrel monotherapy at a dose of 3.75 mg/day with a DAPT prasugrel-based strategy (aspirin 81–100 mg/day and prasugrel 3.75 mg/day) in patients undergoing PCI or ACS. Of the patients enrolled, 75% presented with ACS. The prasugrel monotherapy was not superior to DAPT for major bleeding events (BARC 3 or 5) and, while the cardiovascular events met the non-inferiority criteria, they were more frequent in the monotherapy group within the first 30 days. These findings suggest that a de-escalation strategy immediately following PCI is not advantageous and may, in fact, be harmful, particularly for patients with acute coronary syndrome (ACS) [126]. 

However, while the no-DAPT strategy appeared feasible and could represent a solution for patients at extremely HBR or truly intolerant to aspirin, larger randomized studies are needed before broader clinical implementation. 

## 5. Drug–Drug Interaction

Oral antiplatelet drugs require the activity of CYP enzymes, either for activation (clopidogrel and prasugrel, as they are pro-drugs) or metabolism (ticagrelor); thus, co-administration of inhibitors or inducers of CYP enzymes may result in interactions with antiplatelet drugs [127,128]. P2Y12 receptor inhibitors also, additionally or synergistically, interact with drugs that affect platelet function, such as selective serotonin reuptake inhibitors (SSRIs) [129]. However, the clinical impact of SSRI therapy on patient outcomes based on antiplatelet therapy is likely limited [130]. The drugs that are most commonly used in patients suffering an ACS or undergoing PCI are: statins, beta blockers, angiotensin-converting-enzyme (ACE) inhibitors, angiotensin II receptor blockers (ARBs), calcium channel blockers, proton pump inhibitors (PPIs) and ibuprofen [131]. 

In the PPI family, omeprazole and esomeprazole are potent inhibitors of CYP2C19. In vitro studies suggested a possible interaction of these molecules that leads to a potential reduction in clopidogrel’s efficacy, above all in low-responders [132,133]. For this reason, international guidelines highlight that the association of these drugs might best be avoided [134]. Nonetheless, the clinical impact of such interaction is likely to be limited. Large, randomized control studies evaluating the impact of omeprazole vs. placebo in patients treated with DAPT after coronary stenting demonstrated no excess of ischemic events in patients assigned to omeprazole, and a significant reduction in gastrointestinal events including bleeding [135]. 

In a subgroup analysis of the PRODIGY trial, from Gargiulo et al., evaluating the effect of co-administration of PPI and DAPT, the primary efficacy endpoint did not differ between PPI and no-PPI users (9.2% vs. 11.5%; adj. HR: 1.051; 95% confidence interval [CI] 0.788–1.400; *p* = 0.736); bleeding rates and NACE were also similar, suggesting that, when indicated, concomitant use of PPI and clopidogrel is not associated with adverse clinical outcomes [64].

Another drug that has the potential to cause pharmacodynamic interaction with antiplatelet drugs is ibuprofen, increasing the bleeding risk. In general, in a nationwide study from Denmark, it was demonstrated that the use of NSAIDs together with antithrombotic drugs increases the bleeding risk [136]. Long-term use of NSAIDS or corticosteroids is considered an HBR criterion, and the association of these drugs should be avoided owing to the increased bleeding risk [137]. 

The potential interaction between antiretroviral medications and antiplatelet agents has been a subject of investigation in multiple studies. As mentioned earlier, drugs like prasugrel and clopidogrel rely on specific enzymes, including cytochrome P450 (CYP) 3A4, CYP2C19, and CYP2B6, for their bioactivation. Ritonavir and cobicistat are potent inhibitors of CYP3A that play a crucial role in the treatment of human immunodeficiency virus (HIV) infection. Research has shown that HIV-infected patients receiving boosted anti-retroviral therapies exhibit significantly reduced exposure to clopidogrel and prasugrel’s active metabolites compared to healthy individuals. Notably, clopidogrel failed to provide adequate platelet inhibition in 44% of HIV patients, while prasugrel achieved robust platelet inhibition in both healthy subjects and those with HIV [138]. Despite these concerns about pharmacokinetic interactions, the clinical implications remain incompletely understood. It is imperative to conduct further research to gain a better understanding of the scope and clinical relevance of these interactions and to establish evidence-based guidelines for managing cardiovascular events in this unique patient population. 

In conclusion, drug–drug interaction is a relevant factor to consider while choosing DAPT strategy—however, not in terms of duration, but in terms of which P2Y12 receptor inhibitor is prescribed.

## 6. Conclusions

DAPT has been the cornerstone of treatment after coronary stenting and secondary prevention after an ACS. Individualized antiplatelet therapy after stenting represents an opportunity to balance ischemic and bleeding risks, especially in higher-risk patients. Procedural complexity, patient characteristics and bleeding risk collectively shape the decision-making process surrounding DAPT. The quest for a ‘one-size-fits-all’ regimen is progressively supplanted by the recognition of a nuanced, patient-centered approach.
